# Reticulated Retinoic Acid Synthesis is Implicated in the Pathogenesis of Dry Eye in Aqp5 Deficiency Mice

**DOI:** 10.1167/iovs.65.8.25

**Published:** 2024-07-17

**Authors:** Huanhuan Ge, Guohu Di, Bin Li, Wenshuo Han, Peirong Song, Shiheng Han, Dianqiang Wang, Peng Chen

**Affiliations:** 1 School of Basic Medicine, Qingdao University, Qingdao, China; 2 Aier School Ophthalmology, Central South University, Changsha, Hunan, P. R. China; 3 Department of Ophthalmology, Qingdao Aier Eye Hospital, Qingdao, China; 4 Institute of Stem Cell Regeneration Medicine, School of Basic Medicine, Qingdao University, Qingdao, China

**Keywords:** aquaporin 5 (AQP5), retinoic acid (RA), dry eye disease (DED)

## Abstract

**Purpose:**

Abnormalities in aquaporins are implicated in the pathological progression of dry eye syndrome. Retinoic acid (RA) regulates cellular proliferation, differentiation, and apoptosis in the cornea, thereby being associated with dry eye disease (DED). The objective of this study is to explore the underlying mechanisms responsible for RA metabolic abnormalities in corneas lacking aquaporin 5 (AQP5).

**Methods:**

Dry eye (DE) models were induced via subcutaneous scopolamine hydrobromide. Aqp5 knockout (*Aqp5*^−/−^) mice and DE mice were utilized to assess corneal epithelial alterations. Tear secretion, goblet cell counts, and corneal punctate defects were evaluated. The impact of Aqp5 on RA-related enzymes and receptors was investigated using pharmacological RA or SR (A JunB inhibitor), a transcription factor JunB inhibitor, treatment in mouse corneal epithelial cells (CECs), or human corneal epithelial cells (HCECs). The HCECs and NaCl-treated HCECs underwent quantitative real-time PCR (qRT-PCR), immunofluorescent, Western blot, and TUNEL assays. The regulation of transcription factor JunB on Aldh1a1 was explored via ChIP-PCR.

**Results:**

Aqp5 and Aldh1a1 were reduced in both CECs of DE mice and NaCl-induced HCECs. *Aqp5^−/−^* mice exhibited DE phenotype and reduced Aldh1a1. RA treatment reduced apoptosis, promoted proliferation, and improved the DE phenotype in *Aqp5^−/−^* mice. JunB enrichment in the Aldh1a1 promoter was identified by ChIP-PCR. SR significantly increased Aldh1a1 expression, Ki67, and ΔNp63-positive cells, and decreased TUNEL-positive cells in CECs and HCECs.

**Conclusions:**

Our findings demonstrated the downregulation of Aqp5 expression and aberrant RA metabolism in DE conditions. Knockout of Aqp5 resulted in reduced production of RA through activation of JunB, subsequently leading to the manifestation of DE symptoms.

The multifactorial ocular disease known as dry eye disease (DED) significantly impairs quality of life in adulthood, manifesting with symptoms such as ocular dryness, burning sensation, pain, and light sensitivity.^1^ The ocular surface is covered by corneal and conjunctival epithelia, and any distortions to it can result in the development of DED.^1^^,^^2^ A defining characteristic of this condition is tear film hyperosmolarity, which triggers the production of inflammatory mediators that can damage goblet cells and ocular epithelium, disrupting the tear film and ultimately resulting in DED.^3^ The middle aqueous layer contains vitamin A, known as retinol, which serves as a nutrient for the corneas and promotes wound healing.^4^ Deficiency of vitamin A or its metabolites is considered an important pathophysiologic mechanism in various diseases, including xerophthalmia and DED.^5^ However, the regulation mechanism of vitamin A in the corneal epithelium has not been fully investigated in DED.

Aquaporins constitute a small family of transmembrane proteins that facilitate osmotically driven water transport across the cytoplasmic membrane. Hydrogen peroxide (H_2_O_2_) is one of the most abundant and persistent reactive oxygen species (ROS) molecules and has been discovered as a novel kind of substrate for members of the aquaporin superfamily.^6^^,^^7^ Aquaporin 5 (AQP5) proteins are significantly expressed in the plasma membrane of the corneal epithelium.^8^^,^^9^ Transgenic knockout mouse research has provided insights into the role of aquaporins in impaired salivary and submucosal gland secretion.^10^^,^^11^ Studies on mice with Aqp5 deficiency have reported an increase in corneal thickness and a decrease in osmotic water permeability across the corneal epithelium.^12^ The recent findings also suggest that Aqp5 can augment cellular migration and proliferation, thereby expediting corneal wound healing.^13^ Collectively, these findings offer compelling evidence to support the indispensable role of AQP5 in maintaining ocular homeostasis and normal function within ocular surface tissues, with aberrations in AQP5 potentially serving as a pivotal factor contributing to DED.

The presence of retinol in the precorneal tear film is crucial for maintaining the homeostasis of the ocular surface.^14^ Retinol, once internalized by corneal epithelial cells (CECs), undergoes two sequential oxidative steps: first converting into retinal and then further metabolizing into retinoic acid (RA) through the catalytic actions of retinol dehydrogenases (RDHs) and aldehyde dehydrogenases (ALDHs).^15^^,^^16^ As a biologically active form of retinol, RA plays a crucial role as a signaling molecule in coordinating various physiological processes through its binding to RA receptor alpha, beta, and gamma (Rar α, β, and γ).^17^ Excessive RA is metabolized into inactive compounds by Cytochrome P450 family 26 subfamily B member 1 (Cyp26b1).

The deficiency of vitamin A has long been recognized for its ability to induce abnormal differentiation of the ocular surface, resulting in ulceration, epithelial squamous metaplasia, keratinization of the cornea and conjunctiva, and loss of goblet cells in the conjunctiva.^18^ The AP-1 transcription factor family comprises Jun (c-Jun, JunB, and JunD), Fos, and ATF. Through the formation of homodimers or heterodimers and their binding to DNA via the DNA binding motif, these factors exert influence over the target genes' transcription. Other researchers have demonstrated that Aldh1a1 in the mouse liver is regulated by AP-1, which can be recruited by mitogen-activated protein kinases.^19^

Our earlier research has shown that the knockout of Aqp5 leads to defects in corneal epithelium^20^ and abnormalities in homeostasis.^21^ Studies have demonstrated that RA therapy maintains the integrity of the epithelial barrier, thereby regulating the proliferation and migration of CECs^22^ and facilitating cell adhesion during corneal epithelium repair.^23^ Furthermore, emerging evidence suggests that all trans-RA provides its anti-inflammatory actions by suppressing the production of pro-inflammatory chemicals (interleukin-1β [IL-1β], IL-12, and RANTES) associated with DE while enhancing the generation of IL-10, an anti-inflammatory cytokine.^15^^,^^24^

In this study, we observed a downregulation of Aqp5 and Aldh1a1 expression, as well as an upregulation of JunB expression in the CECs of DE-induced female C57BL/6 (*Aqp5^+/+^* DE) and Aqp5 knockout (*Aqp5*^−/−^) mice. Through RNA sequencing and ChIP-PCR assays, we discovered that JunB combined with and regulated Aldh1a1. Inhibition of JunB or application of RA effectively reduced apoptosis and promoted proliferation in the corneal epithelium of DE mice and NaCl-induced human corneal epithelial cells (HCECs). These findings suggest that Aqp5 is involved in RA synthesis through the transcription factor JunB, with its deficiency leading to DE.

## Materials and Methods

### Animal Models and Treatment

*Aqp5* ^−/−^ mice were obtained according to the previously described method.^25^ All animal experiments were conducted with the approval of Qingdao University’s Experimental Animal Ethics Committee (approval ID: QDU-AEC-2024466) and in strict adherence to the guidelines outlined in the ARVO Statement. Experimental DE models were induced in *Aqp5^+/+^* mice (6-month-old mice, *n* = 50) by subcutaneously injecting scopolamine hydrobromide (Meilubio) 4 times daily for 5 consecutive days. SR is a JunB inhibitor. The *Aqp5^+/+^* DE+SR mice (*n* = 15) were subconjunctivally injected with 3 µL of SR (10 mM/L; TargetMol) at 24 hours before, 24 hours after, and 72 hours after the initiation of DE induction. The *Aqp5^−/−^*+RA (*n* = 15) or *Aqp5^−/−^* +SR mice (*n* = 15) received subconjunctival injections of 3 µL RA (1 mM/L; TargetMol) or SR (10 mM/L), respectively, once every other day for a total of 2 times.

The lateral canthus of mice was exposed to a cotton thread soaked in phenol for a duration of 20 seconds. Tear secretion was quantified by measuring the length of moisture in millimeters. The conjunctival sac was stained with 0.25% fluorescein sodium solution for corneal fluorescein staining. Subsequently, the sample was captured using a cobalt blue light and recorded on camera.

### Cell Culture and Treatment

The immortalized HCEC line was cultured as previously described.^26^ Hyperosmolality-induced HCECs were established by exposing them to a 110 mM NaCl solution. Prior to collection for Western blot (WB) and immunofluorescence (IF) staining analysis, the medium was supplemented with either RA or SR at a final concentration of 1 µM or 10 µM, respectively, for a duration of 24 hours. Acetazolamide (AZA) was added to the culture medium until reaching a final concentration of 10 mM.

### RNA Sequencing

The CECs of *Aqp5^+/+^* and *Aqp5^−/−^* mice were used for total RNA extraction. Subsequently, high-throughput sequencing of the extracted RNA was performed following a previously established protocol.^21^ In brief, the NEBNext rRNA Depletion Kit (New England Biolabs) was used according to the manufacturer’s instructions to remove rRNAs from the total RNA sample. The NEBNext UltraTM II Directional RNA Library Prep Kit (New England Biolabs) was utilized to generate RNA libraries as per the manufacturer’s guidelines. Library sequencing was conducted using an Illumina Novoseq device with 150 bp paired-end reads.

The paired-end reads extracted from the Illumina Noveseq 6000 sequencer were subjected to quality control using Q30. The high-quality clean reads were then aligned to the reference genome using Hisat2 (version 2.0.4) after 3′ adaptor-trimming and removal of low-quality reads with Cutadapt (version 1.9.3). Normalization of raw counts was performed using edgeR, followed by identification of differentially expressed mRNAs based on *P* value and fold change thresholds. HTSeq software (version 0.9.1) was utilized for obtaining this information, with a threshold of *P* value ≤ 0.05 and fold change ≥2 for determining differences.

### Quantitative Real-Time PCR 

A FastPure Cell/Tissue Total RNA Isolation Kit V2 (Vazyme) was used for the extraction of total RNA from the CECs and HCECs. Reverse transcription of the aforementioned RNA samples was performed using a PrimeScript RT Reagent Kit (TaKaRa) to generate cDNA. SYBR Green reagents (Vazyme) were utilized for quantitative real-time PCR (qRT-PCR). The comparative threshold cycle approach, with GAPDH as an endogenous reference gene, was used to evaluate the PCR results. The Table includes a comprehensive list of all primer sequences used in this investigation (see the Table).

**Table.** Gene-Specific Primers Used in This Study

Gene Name	Primer Type	Primer Sequence	Application
m-Aldh1a1	Forward	TTTTCTGAGTGGCATTTGTTAGCA	ChIP-PCR
	Reverse	GGCACCACCACTGTGCAA	
m-Aldh1a1	Forward	CTGCAGGGAAAAGCAATCTGA	q-PCR
	Reverse	ATGCTGCGACACAACATTGG	
m-Aqp5	Forward	GCGCTCAGCAACAACACAAC	
	Reverse	GTGTGACCGACAAGCCAATG	
m-JunB	Forward	CTGTGTCCCCCATCAACATG	
	Reverse	GCGTTCTCAGCCTTGAGTGTCT	
m-c-Jun	Forward	CCCCTATCGACATGGAGTCTCA	
	Reverse	CGGAGTTTTGCGCTTTCAAG	
m-JunD	Forward	GTCGCCCATCGACATGGA	
	Reverse	CTCGGTGTTCTGGCTTTTGAG	
m-Rarα	Forward	GCTGCTGGAAGCACTGAAAGT	
	Reverse	CCTGGGATCTCCATCTTCAATG	
m-Cyp26b1	Forward	GCGCTACCTGGACTGTGTCA	
	Reverse	TCGTGAGTGTCTCGGATGCTA	
h-Aldh1a1	Forward	TGCAGGTTGGGCTGACAA	
	Reverse	GCAGGCCCTATCTTCCAAATG	
h-Aqp5	Forward	GGTGGTCATGAATCGGTTCAG	
	Reverse	GGCTCATACGTGCCTTTGATG	
h-Rarα	Forward	CTCATTGAGAAGGTGCGCAAA	
	Reverse	GATGCACTTGGTGGAGAGTTCA	
h-Cyp26b1	Forward	CAGCAGTTTGTGGACAATGTCTTC	
	Reverse	CCAAGTAGTCCTTGCCCTGTGT	

### WB Analysis

The CECs and HCECs were utilized for protein extraction. The samples were thoroughly mixed in a chilled RIPA buffer. SDS-PAGE was used to separate and quantify denatured total proteins, which were subsequently electronically transferred onto PVDF membranes (Millipore). Subsequently, the blots were incubated with primary antibodies against β-actin (1:1000; ABclonal), Aqp5 (1:1000; ABcolonal), Aldh1a1 (1:1000; ABclonal), JunB (1:500; ABclonal), Bcl-2 (1:1000; Abcam), Bax (1:500; Affinity), and cleaved caspase-3 (1:500; Cell Signaling Technology) for 1.5 hours while being blocked with 5% skim milk. An hour was spent incubating the corresponding second antibody. An automated system for chemiluminescence image analysis was used to take the pictures.

### IF Staining

Eye samples were collected from euthanized mice, fixed in optimal cutting temperature compound (OCT), and sectioned into 7 µm frozen sections. Corneal sections and HCECs were then incubated in 4% paraformaldehyde (PFA) for 20 minutes before undergoing membrane disruption with 1% Triton X-100. The samples were incubated with Aqp5 (1:200; Santa), Aldh1a1 (1:200; ABcolonal), Ki67 (1:200; Abcam), and ΔNp63 (1:200; BioLegend) for 1.5 hours and subsequently incubated with secondary antibodies for 1 hour. The sections and cells were imaged using a fluorescence microscope (Olympus BX50).

Terminal Deoxynucleotidyl Transferase mediated dUTP Nick-End Labeling (TUNEL) was conducted according to the instructions of the manufacturer (YEASEN).

### Immunohistochemistry and Periodic Acid-Schiff Staining 

After fixation in 4% PFA, the eyeballs were embedded in paraffin and sectioned into 7 µm slices. Following deparaffinization and antigen retrieval, tissue sections were subjected to immunostaining using cleaved caspase-3 antibodies (1:200; Cell Signaling Technology) and visualized under a fluorescence microscope. Periodic Acid-Schiff (PAS) staining was carried out in accordance with the staining kit’s manufacturer’s instructions (Servicebio). Light microscopy images were captured for each section.

### Scanning Electron Microscopy 

The fresh corneal tissue chunks were harvested within 1 to 3 minutes, and their size did not exceed 5 mm^2^. Subsequently, the tissue blocks were washed and promptly fixed in electron microscopy fixative (ServiceBio) for a duration of 2 hours at ambient temperature, followed by transfer to 4°C for preservation. After a post-fixation step with 1% OsO4 for 1 to 2 hours at room temperature and subsequent gradient ethanol dehydration, the samples underwent drying using a critical point dryer. Finally, all samples were affixed to metallic stubs and coated with gold using sputter-coating equipment (MC1000; HITACHI) for a period of 30 seconds prior to observation.

### Colony Formation Unit Assay

The T25 tissue culture flasks were initially seeded with 3000 cells in DMEM/F12 supplemented with 10% fetal bovine serum and cultured for a duration of 3 days. Subsequently, the culture medium was replaced, and 1 uM RA, 10 uM SR, 10 mM AZA, or 70 mM NaCl were added. Following an additional incubation period of 5 days, the colonies were fixed for 20 minutes using a solution containing 4% PFA and subsequently stained with a solution of 1% crystal violet for a duration of 10 minutes.

### ChIP Assay

The BeyoChIPTM Enzymatic ChIP Assay Kit (Beyotime) was used for the execution of the ChIP assay. In brief, primary CECs were fixed using 1% formaldehyde, followed by glycine addition to neutralize any unreacted formaldehyde. After incubating with Protein A/G Magnetic Beads and Salmon Sperm DNA, crosslinked chromatin (partially set aside as input) was immunoprecipitated using a 2 µg antibody against JunB (C-11) X (Santa Cruz). The supernatant was collected after washing the pellets and treated with 5M NaCl at 65°C for 2 hours to facilitate decrosslinking. Subsequently, purified DNA fragments underwent nested PCR amplification, and the resulting PCR products were subjected to electrophoresis on an agarose gel. The table provides a list of primers utilized in ChIP-PCR.

### Statistical Analyses

The statistical software GraphPad Prism was used for all data analyses. Data between two individual groups were analyzed using a Student’s *t*-test, whereas data among 3 or more groups were compared using a 1-way analysis of variance (ANOVA). The mean ± standard deviation (SD) was presented for the data, and statistical significance was determined at *P* < 0.05.

## Results

### Aberrations of Aqp5 and RA Synthesis in DE Models

**Figure 1.**
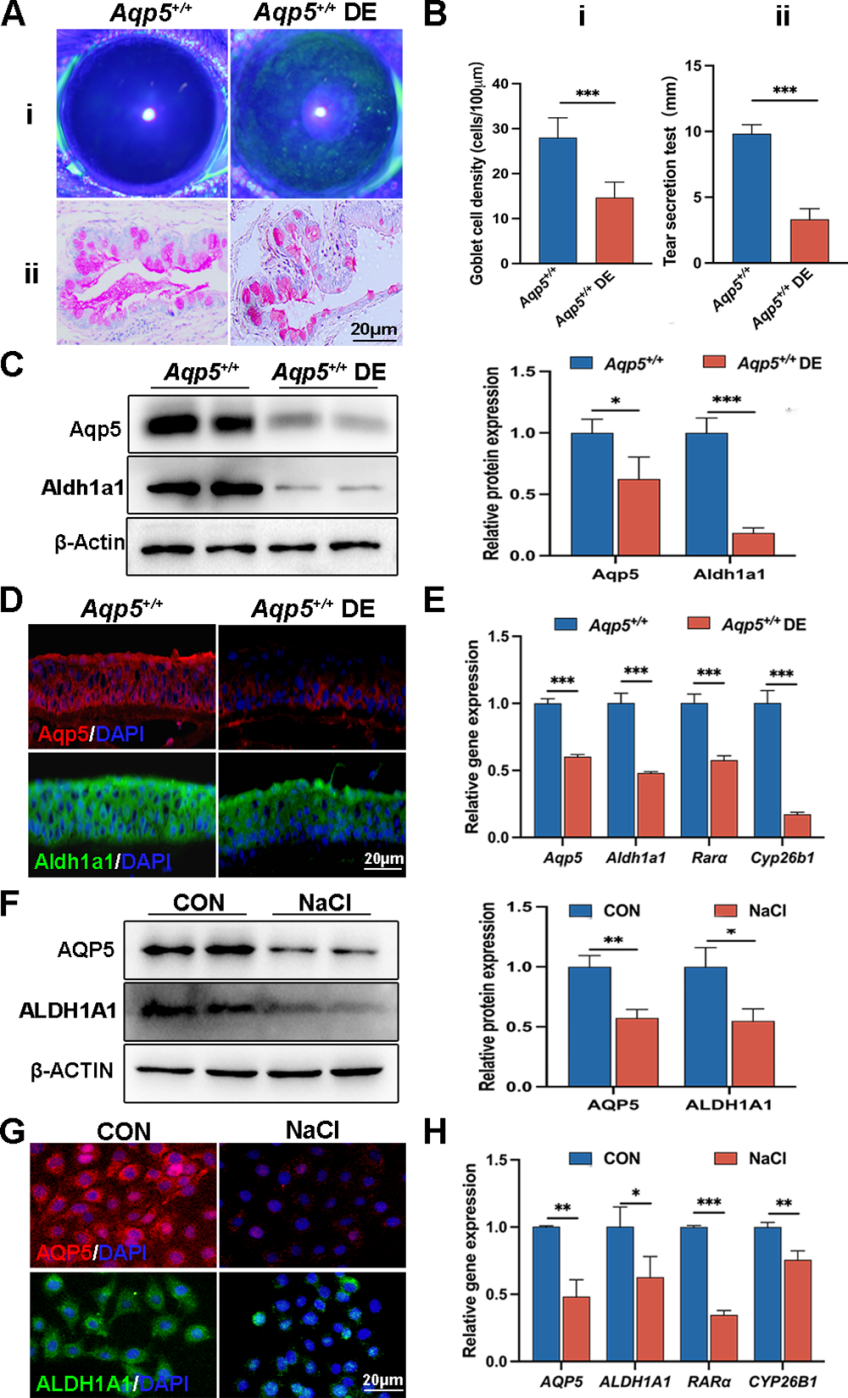
**The abnormal expression of Aqp5 and RA synthesis in DE models.** (**A**) Representative fluorescein staining images (**i**) and PAS staining images (**ii**) of *Aqp5^+/+^* and *Aqp5^+/+^* DE mice. Scale bars = 20 µm. (**B**) Goblet cell density per 100 µm (**i**) and tear secretion test (**ii**) of *Aqp5^+/+^* and *Aqp5^+/+^* DE mice (*n* = 6). (**C**) WB shows the protein levels of Aqp5 and Aldh1a1 in the *Aqp5^+/+^* and *Aqp5^+/+^* DE mice corneas, with β-actin as the internal control (*n* = 3). (**D**) Representative immunofluorescence images of corneal epithelium in *Aqp5^+/+^* and *Aqp5^+/+^* DE mice. Scale bars = 20 µm. (**E**) The qRT-PCR detects the expression of Aqp5 and RA signaling-associated genes (*n* = 3). (**F,** **G**) WB (*n* = 3) and IF show AQP5 and ALDH1A1 expression in HCECs of the control and NaCl groups. Scale bars = 20 µm. (**H**) The qRT-PCR detects AQP5 and RA signaling-associated gene expression in HCECs of the control and NaCl groups (*n* = 3). The statistical analysis results are presented as the mean ± SD. *P < 0.05; **P < 0.01, and ***P < 0.001.

The corneal fluorescein sodium staining revealed distinct punctate staining in the corneas of *Aqp5^+/+^* DE mice (Fig. 1Ai). The conjunctiva of *Aqp5^+/+^* DE mice had 15.71 ± 3.971 goblet cells, which was substantially less than that of the *Aqp5^+/+^* group, according to PAS staining data (28.00 ± 4.427, *P* < 0.001; Figs. 1Aii, Bi). Meanwhile, tear production quantification demonstrated a significant reduction following DE induction compared to *Aqp5^+/+^* mice (Fig. 1Bii). Furthermore, reduced Aqp5 and Aldh1a1 protein levels were seen in the *Aqp5^+/+^* DE corneas by WB (Aqp5: *Aqp5^+/+^* 1.000 ± 0.1119; *Aqp5^+/+^* DE 0.6252 ± 0.1785; Aldh1a1: *Aqp5^+/+^* 1.000 ± 0.1221; *Aqp5^+/+^* DE 0.1853 ± 0.04113; Fig. 1C), which were also confirmed by IF (Fig. 1D). In *Aqp5^+/+^* DE mice, the corneas exhibited significantly reduced mRNA expression levels of Aqp5, Aldh1a1, Rarα, and Cyp26b1 (Fig. 1E). Hypertonic NaCl-induced HCECs demonstrated lower levels of AQP5 and ALDH1A1 expression (Figs. 1F, 1G). The qRT-PCR analysis of HCECs revealed decreased expression of genes involved in the RA pathway in the NaCl group compared to the control group (Fig. 1H). The findings imply a dysfunction in RA synthesis and a reduction in AQP5 expression within the corneal epithelium with DE.

### The Deficiency of Aqp5 Resulted in DE and Perturbation of the RA Pathway

**Figure 2.**
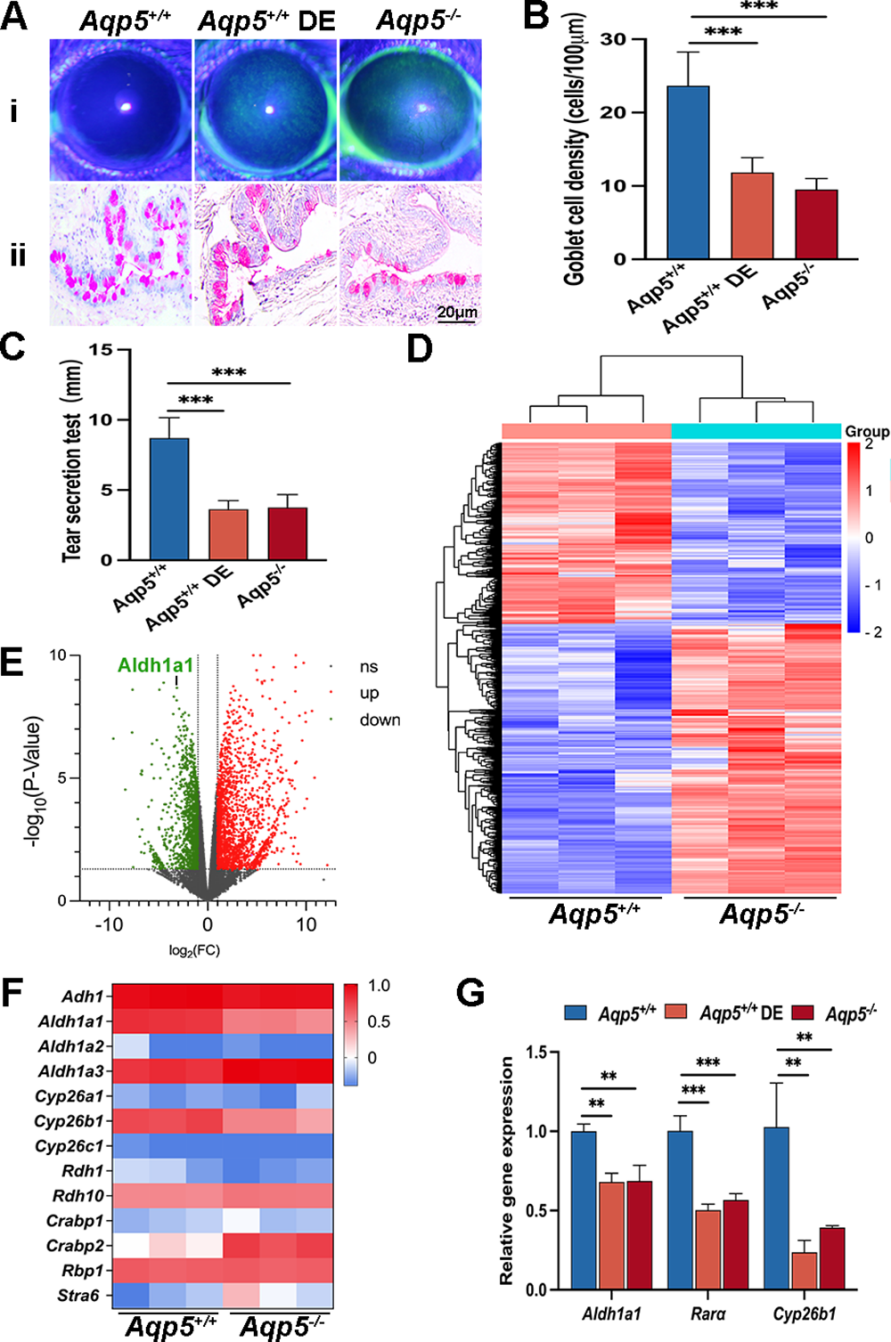
**Aqp5 deficiency causes DE phenotypes and suppresses Aldh1a1 expression.** (**A**) Representative images of corneal fluorescein staining (**i**) and PAS staining (**ii**). Scale bars = 20 µm. (**B**) Goblet cell density per 100 µm (*n* = 6). (**C**) Quantification of the tear secretion test (*n* = 10). (**D**) Heatmap comparison of the differentially expressed genes between *Aqp5^+/+^* and *Aqp5^−/−^* corneal epithelium. (**E**) Volcano plot of the significant differentially expressed genes between *Aqp5^+/+^* and *Aqp5^−/−^* corneal epithelium, presented in *red* (*up*) and *green* (*down*). (**F**) A heatmap of differentially expressed mRNA profiles for RA signaling-related genes. (**G**) The qRT-PCR expression analysis of RA-related genes in the *Aqp5^−/−^* corneal epithelium compared to the *Aqp5^+/+^* (*n* = 3), **P < 0.01 and ***P < 0.001.

To investigate the role of Aqp5 in the DE process, we generated *Aqp5^−/−^* mice for experimental purposes. The results obtained from fluorescein sodium staining (Fig. 2Ai), PAS staining (Figs. 2Aii, 2B), and tear production testing (Fig. 2C) collectively indicated that *Aqp5^−/−^* mice exhibited a DE phenotype. In order to gain further insights into the mechanisms underlying the corneal abnormalities caused by Aqp5 deletion, we extracted mRNA from the CECs of both *Aqp5^+/+^* and *Aqp5^−/−^* mice and conducted RNA sequencing analysis. Aqp5 ablation resulted in a significant alteration in the gene expression profiles of CECs (Fig. 2D). The volcano map illustrated the differential gene expression in the CECs of *Aqp5^+/+^* and *Aqp5^−/−^* mice (Fig. 2E). Moreover, heatmap analysis revealed significantly lower expression levels of Cyp26b1 (FC: 6.831, *P* < 0.001) and Aldh1a1 (FC: 8.805, *P* < 0.001) in the CECs of *Aqp5^−/−^* mice when compared to *Aqp5^+/+^* mice (Fig. 2F). The qRT-PCR results showed that the corneas of *Aqp5^−/−^* and *Aqp5^+/+^* DE mice had considerably lower levels of Aldh1a1, Cyp26b1, and Rarα than the equivalent *Aqp5^+/+^* group (Fig. 2G). The above results demonstrate that the ablation of Aqp5 resulted in a significant decrease in the expression of the RA synthesis enzyme Aldh1a1, leading to an inactive state of the RA-Rara pathway.

### The Rescue Effect of RA on Corneal Defects Induced by Aqp5 Deletion

**Figure 3.**
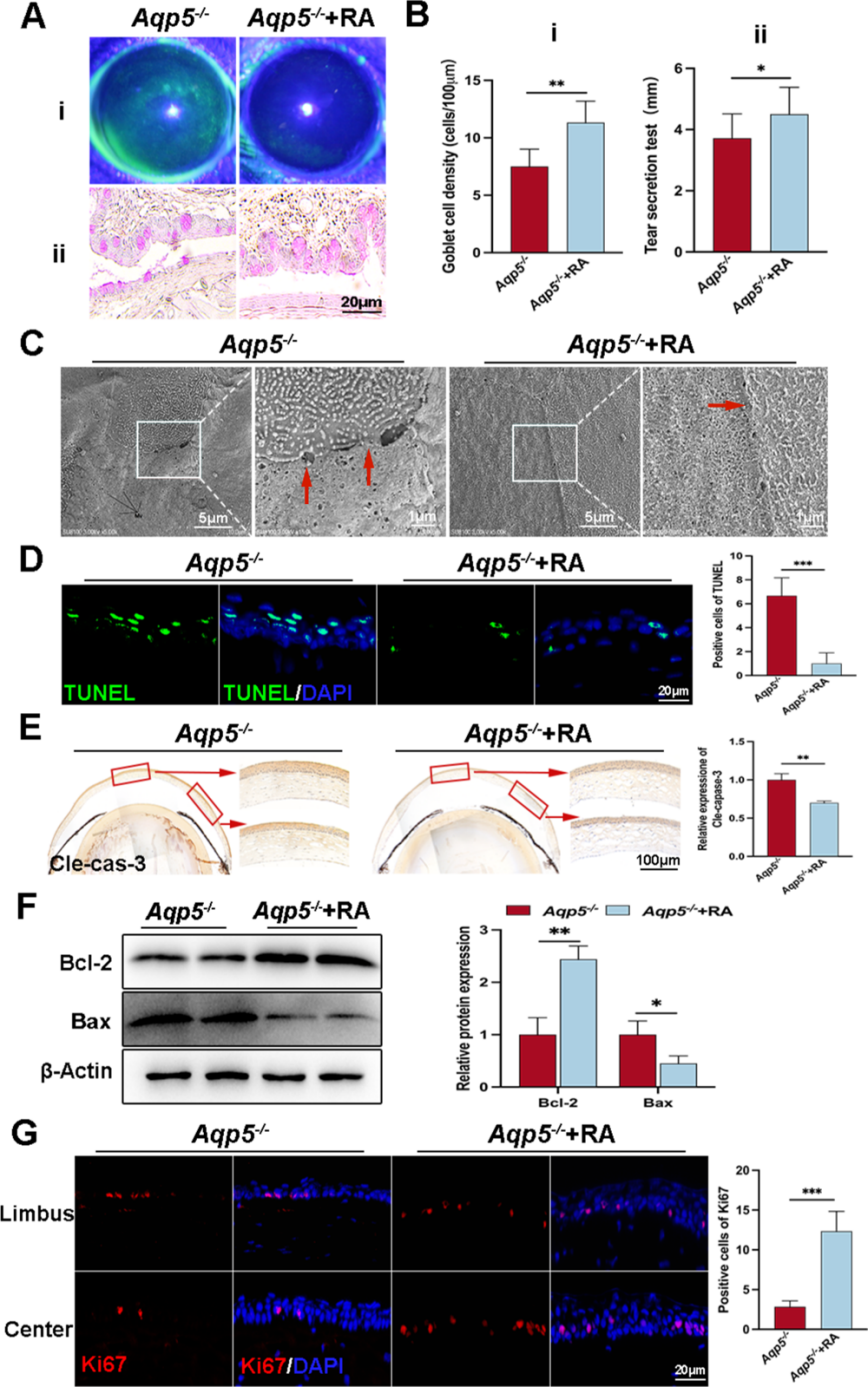
**RA improves the phenotype defects caused by Aqp5 deficiency.** (**A**) Corneal fluorescein staining (**i**) and PAS staining (**ii**) after RA treatment. (**B**) Goblet cell density per 100 µm (**i**) and tear production measurement results (**ii**) (*n* = 6). (**C**) SEM images of the corneas highlight differences in intercellular space, microvilli, and tight junctions between *Aqp5^−/−^* and *Aqp5^−/−^* mice treated with RA. The *right panel* displays high-magnification pictures of the box-selected areas. Scale bars = 5 µm and 1 µm. (**D**) Representative photographs of TUNEL staining in the corneal epithelium of *Aqp5^−/−^* and *Aqp5^−/−^*+RA mice. Scale bars = 20 µm. Statistical analysis is shown in the *right panel* (*n* = 6). (**E**) IHC images of cleaved caspase-3. Scale bars = 100 µm. The quantification results of cleaved caspase-3 positive staining areas in the corneal epithelium are displayed in the *right panel* (*n* = 3). (**F**) WB analysis of Bcl-2 and Bax in the corneal epithelium (*n* = 3). (**G**) IF staining of Ki67 and a positive analysis are presented. Scale bars = 20 µm (*n* = 6). The data are shown as mean ± SD. *P < 0.05, **P < 0.01, and ***P < 0.001.

To investigate the potential role of RA deficiency in DE development in *Aqp5^−/−^* mice, we administered subconjunctival injections of RA to these mice. Remarkably, RA treatment significantly ameliorated corneal defects (Fig. 3Ai), restored goblet cell quantity (Figs. 3Aii, 3Bi), and notably increased tear production volume, as evidenced by the results of the tear secretion test (Fig. 3Bii). Furthermore, scanning electron microscopy (SEM) revealed a restoration of normal corneal epithelial structures, well-organized tight intercellular junctions, and reduced intercellular gaps in *Aqp5^−/−^*+RA mice (Fig. 3C). Comparing *Aqp5^−/−^*+RA to *Aqp5^−/−^* mice, the quantity of apoptotic cells in the corneal epithelium was much lower, going from 6.667 ± 1.506 to 1.0000 ± 0.8944 (Fig. 3D). The expression of cleaved caspase-3 was markedly downregulated, as evidenced by immunohistochemistry (IHC) staining (*Aqp5^−/−^*: 1.000 ± 0.0804; *Aqp5^−/−^*+RA: 0.7000 ± 0.0244, *P* < 0.01; Fig. 3E). The WB analysis revealed a significant inhibition of Bax expression in mouse CECs upon treatment with RA (*Aqp5^−/−^* 1.000 ± 0.2624; *Aqp5^−/−^*+RA 0.4506 ± 0.1433; *P* < 0.05; Fig. 3F). Conversely, RA treatment resulted in an elevation of Bcl-2 protein expression (*Aqp5^−/−^* 1.000 ± 0.3242; *Aqp5^−/−^*+RA 2.441 ± 0.2524, *P* < 0.01; see Fig. 3F). Statistical examination revealed a noteworthy rise of around three times in the quantity of Ki67-positive cells in the RA-treated corneas in contrast to the control group (Fig. 3G). The findings suggest that the defects in the corneas are mediated by a reduction in RA synthesis, and administration of RA could restore the integrity and homeostasis of the epithelial layer disrupted by Aqp5 deletion.

### RA Rescued Defects in HCECs Induced by NaCl

**Figure 4.**
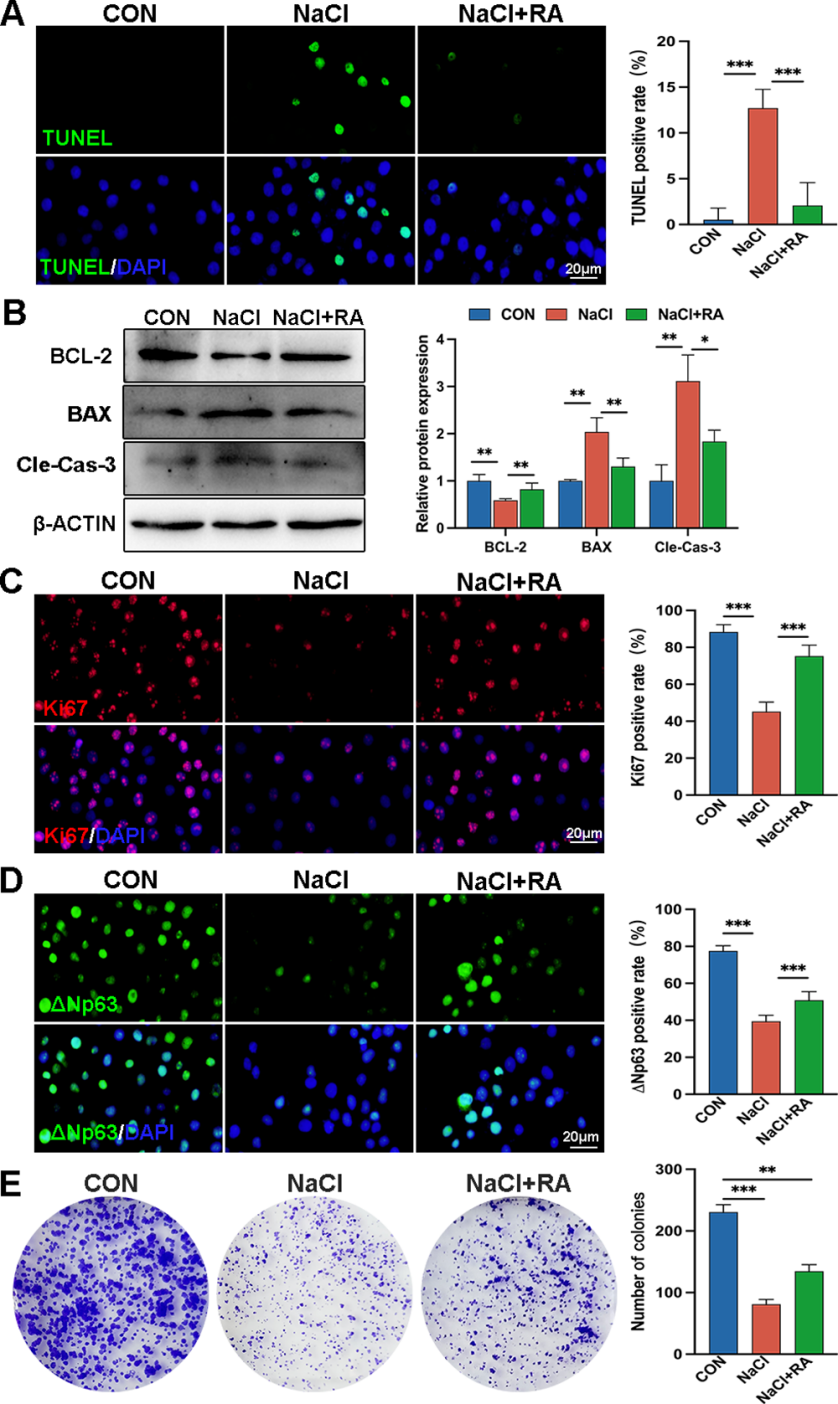
**RA improves the function defects caused by NaCl induction.** (**A**) IF staining of TUNEL-positive cells under hypertonic NaCl induction or co-treatment with RA. The *right panel* presents the analysis results (*n* = 6). Scale bars = 20 µm. (**B**) WB detects the protein expression of BCL-2, BAX, and cleaved caspase-3 in the HCECs (*n* = 3). (**C**) IF images of HCECs labeled with Ki67 (*red*), and the positive analysis is presented in the *right panel* (*n* = 6). Scale bars = 20 µm. (**D**) IF staining of ΔNp63 (*green*) and positive analysis are displayed in the *right panel* (*n* = 6). Scale bars = 20 µm. (**E**) A colony formation assay was performed to demonstrate the proliferation ability of HCECs after treatment with NaCl alone or treatment with RA both. The statistical results of the colonies are shown as the mean ± SD. *P < 0.05; **P < 0.01, and ***P < 0.001.

We cultured HCECs and divided them into three groups: control, NaCl, and NaCl+RA. Compared to the control group, the NaCl group exhibited a significantly higher TUNEL-positive rate, which was markedly reduced upon exposure to 1 µM RA (the control group = 0.5217 ± 1.278%; the NaCl group = 12.71 ± 2.050%; and the NaCl+RA group = 2.070 ± 2.501%; Fig. 4A). Furthermore, in comparison to the NaCl group, the expression levels of BAX and cleaved caspase-3 were significantly downregulated, whereas BCL-2 was evidently upregulated in the NaCl+RA group (Fig. 4B). The proportion of Ki67 positive cells increased significantly from 45.22% in the NaCl group to 75.30% in the NaCl+RA group (Fig. 4C). In contrast to the NaCl group, the expression level of ΔNp63 was upregulated by nearly 1.3-fold in the NaCl+RA group (the NaCl group = 39.43 ± 2.935% and the NaCl+RA group = 50.89 ± 4.134%; Fig. 4D). The colony formation assay revealed that the control group had an average number of colonies at 230.7, whereas it decreased to only 81.00 in the NaCl group; however, treatment with RA resulted in a significant increase to an average number of colonies at 134.7 compared to the NaCl group (Fig. 4E). The above findings validate the role of RA in mediating the impact of Aqp5 deletion on corneal epithelial cell proliferation, while highlighting the protective effect of RA against hypertonicity-induced cell death.

### Aqp5 Regulated Aldh1a1 Through the Transcription Factor JunB

**Figure 5.**
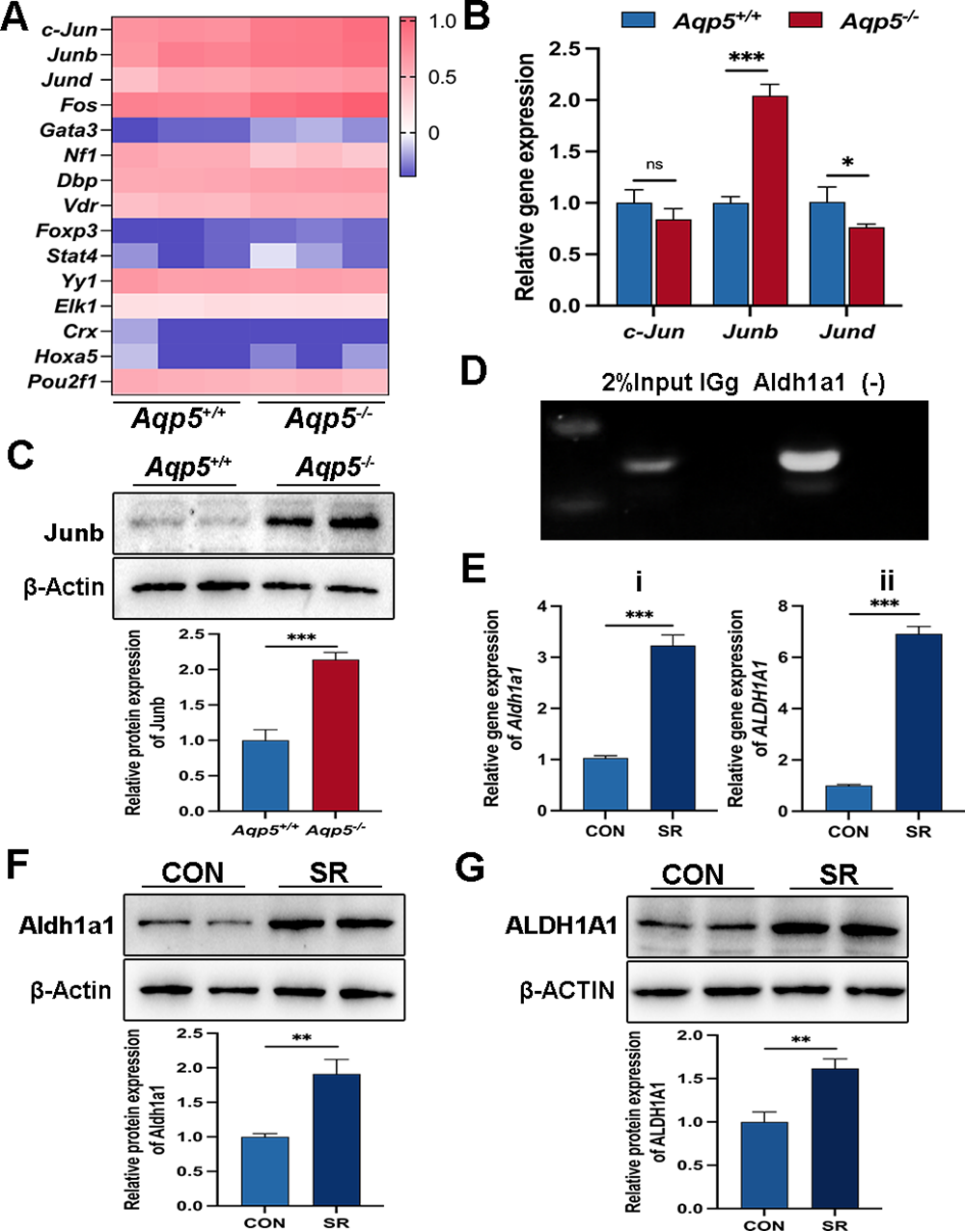
**JunB interacts with Aldh1a1 and modulates the expression of Aldh1a1.** (**A**) Heatmap of transcription factors involved in the transcription of Aldh1a1 in the corneal epithelium of *Aqp5^+/+^* and *Aqp5^−/−^*. (**B**) The qRT-PCR analysis of the expression of c-Jun, JunB, and JunD in the corneal epithelium of *Aqp5^+/+^* and *Aqp5^−/−^* (*n* = 3). (**C**) WB and quantitative analysis show the protein levels of JunB between *Aqp5^+/+^* and *Aqp5^−/−^* mice corneas (*n* = 3). (**D**) ChIP-PCR analysis demonstrates JunB enrichment at the Aldh1a1 promoter. The representative outcome of agarose gel electrophoresis is shown. (**E**) The qRT-PCR shows Aldh1a1 expression levels in cultured primary mouse CECs (**i**) and HCECs (**ii**) after being treated with SR (*n* = 3). (**F**) WB shows Aldh1a1 expression in primary mouse CECs (*n* = 3). (**G**) WB quantitative statistics shows Aldh1a1 expression of HCECs (*n* = 3). All data are presented as mean ± SD. *P < 0.05, **P < 0.01, and ***P < 0.001, and ns, no significance.

To further investigate the underlying mechanisms by which Aqp5 influences Aldh1a1 expression, we utilized the PROMO website (https://alggen.lsi.upc.es/cgibin/promo_v3/promo/promoinit.cgi?dirDB=TF_8.3) to predict the transcription factors (TFs) associated with Aldh1a1. The heatmap visually represents the mRNA expression levels of these TFs (Fig. 5A). *Aqp5^−/−^* mice had twice as much JunB expression in their corneal epithelium as *Aqp5^+/+^* mice, according to a qRT-PCR investigation (Fig. 5B). The WB analysis further confirmed an increased expression of JunB in the corneas of *Aqp5^−/−^* mice compared to *Aqp5^+/+^* mice (Fig. 5C). The ChIP-PCR results provided verification for the presence of a JunB binding site in the promoter region of Aldh1a1 (Fig. 5D). To investigate the regulatory impact of JunB on Aldh1a1 expression levels, we treated primary mouse CECs from *Aqp5^+/+^* mice and HCECs with SR. The qRT-PCR results revealed a significant upregulation in mRNA expression levels of Aldh1a1 after SR treatment (Fig. 5E). Meanwhile, the expression of Aldh1a1 was significantly upregulated, as shown by WB analysis in the primary mouse CECs following SR treatment compared to the control group (Fig. 5F). Notably, HCECs treated with SR exhibited markedly elevated protein levels of Aldh1a1 when compared to the control group (Fig. 5G). The findings suggest that JunB exerts direct control over the expression of Aldh1a1 in CECs.

### SR Mitigated Corneal Epithelial Apoptosis and Alleviated the Symptoms of DE

**Figure 6.**
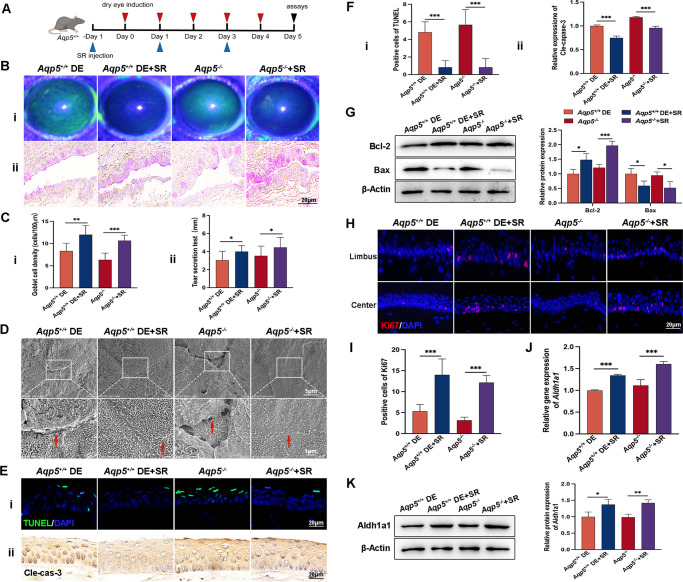
**SR alleviates DE phenotype.** (**A**) Timeline of DE induction in *Aqp5^+/+^* mice and subsequent treatment with SR. (**B**) Representative images of fluorescein sodium staining in the corneas (**i**) and PAS staining in the conjunctiva (**ii**). (**C**) Quantitative analysis of goblet cell density (**i**) and tear production (**ii**) per group (*n* = 6). (**D**) Typical photographs of corneal electron microscopy scanning. Scale bars = 5 µm and 1 µm. (**E**) Representative photographs of TUNEL staining (**i**) and IHC of cleaved caspase-3 (**ii**). Scale bars = 20 µm. (**F**) Statistical analyses of **Ei** (*n* = 6) and **Eii** (*n* = 3), ***P < 0.001. (**G**) WB analysis shows the expression levels of Bcl-2 and Bax in the corneal epithelium (*n* = 3). (**H**) IF staining of Ki67. Scale bars = 20 µm. (**I**) Quantitative statistics of the Ki67 positive rate. (**J**) The qRT-PCR analysis proves the increased expression of Aldh1a1 (*n* = 3). (**K**) WB analysis of Aldh1a1 after treatment with SR in both *Aqp5^+/+^* DE and *Aqp5^−/−^* mice corneas (*n* = 3). All data are presented as mean ± SD. *P < 0.05, **P < 0.01, ***P < 0.001.

To investigate the impact of SR on the DE process, it was used as a treatment for *Aqp5^+/+^* DE and *Aqp5^−/−^* mice. The administration procedure is illustrated in Figure 6A. SR treatment effectively mitigated punctate defects in the corneal epithelium of both *Aqp5^+/+^* DE and *Aqp5^−/−^* mice (Fig. 6Bi). Notably, conjunctival goblet cell density (see Figs. 6Bii, 6Ci) and tear secretion (Fig. 6Cii) exhibited significant increases in the SR treatment group. SEM revealed that SR efficiently ameliorated damaged and dissolved cell membranes while enhancing tight junctions and gaps among CECs in both *Aqp5^+/+^* DE and *Aqp5^−/−^* mice (Fig. 6D). The quantity of TUNEL-positive cells (Fig. 6Ei) and cleaved caspase-3 expression (Fig. 6Eii) were reduced in the group treated with SR; statistical analyses were presented in Figure 6F. WB analysis confirmed that Bax was significantly downregulated, whereas Bcl-2 was markedly upregulated in the SR-treated groups compared to both the *Aqp5^+/+^* DE and *Aqp5^−/−^* groups (Fig. 6G). The SR treatment groups exhibited a notably greater quantity of CECs that were positive for Ki67 staining (Figs. 6H, 6I). In *Aqp5^+/+^* DE and *Aqp5^−/−^* mice, SR treatment directly increased Aldh1a1 expression in the corneal epithelium (Figs. 6J, 6K). The collective findings suggest that Aqp5 plays a crucial role in regulating corneal epithelial homeostasis and regeneration through its promotion of RA synthesis via JunB.

### The Function and Expression of Aldh1a1 in HCECs Were Enhanced by SR

**Figure 7.**
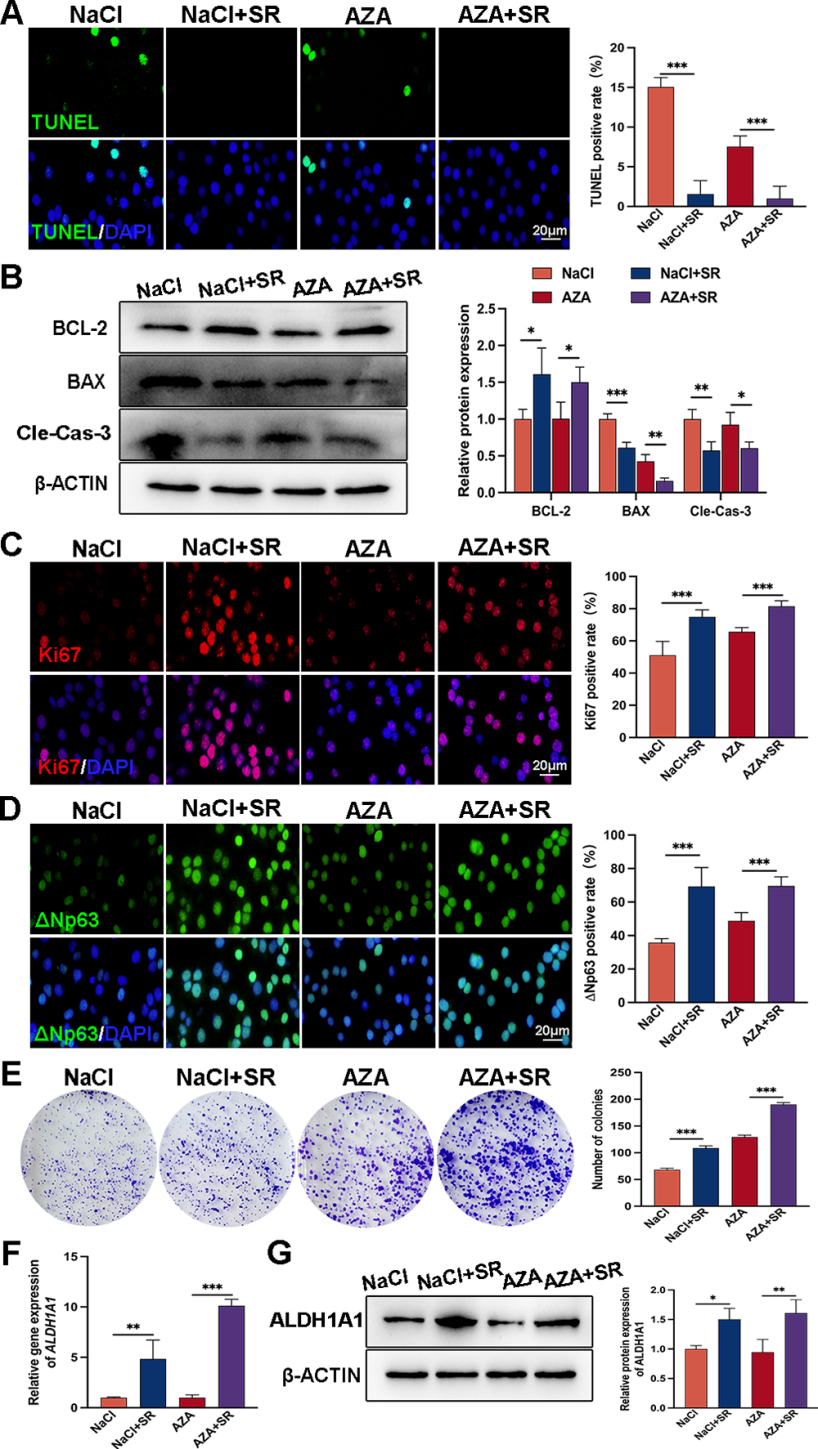
**SR improves the function defects in NaCl or AZA-treated HCEC cells.** (**A**) TUNEL staining and statistical analysis (*n* = 6). Scale bars = 20 µm. (**B**) WB analysis shows the levels of apoptosis-related proteins in HCECs (*n* = 3). (**C**) Representative photographs of Ki67 staining and statistical data (*n* = 6). Scale bars = 20 µm. (**D**) Representative images of ΔNp63 staining and statistical results (*n* = 6). Scale bars = 20 µm. (**E**) A colony formation assay was applied to evaluate the cell proliferation ability after treatment with SR. The right panel presents the analysis results (*n* = 3). (**F,** **G**) The qRT-PCR analysis and WB analysis verifies the elevation of Aldh1a1 after treatment with SR (*n* = 3). All data are presented as mean ± SD. *P < 0.05, **P < 0.01, and ***P < 0.001.

AZA is an inhibitor of aquaporins that exerts its function by downregulating aquaporin expression.^27^ In order to simulate the deletion of Aqp5, HCECs were treated with AZA. Compared to the control group, the number of TUNEL-positive cells in HCECs treated with SR significantly decreased (Fig. 7A). WB analysis revealed a noticeable increase in Bcl-2 expression in HCECs from both the NaCl+SR and AZA+SR groups, compared to the NaCl and AZA groups, respectively, whereas levels of Bax and cleaved caspase-3 were reduced (Fig. 7B). Moreover, there was a significant increase in Ki67 and ΔNp63 positive cells following SR treatment (Figs. 7C, 7D). Furthermore, colony-forming assays demonstrated enhanced proliferation activity after SR application, as evidenced by a notable increase in the number of cell clones (Fig. 7E). To investigate whether Aldh1a1 played a role in this change, we examined alterations in its expression levels. The mRNA and protein expression levels of Aldh1a1 were higher in the SR-treated groups than those treated with NaCl or AZA (Figs. 7F, 7G). The in vitro findings further validate the involvement of JunB in the interplay between AQP5 and RA synthesis.

## Discussion

The pathogenesis of DED encompasses disorders related to tear film instability and hyperosmolarity, dysfunction in the neurological system, inflammation of the ocular surface, and injury.^28^ The impairment of the corneal epithelial barrier is regarded as a crucial pathophysiological mechanism underlying DED.^29^^,^^30^ Current therapeutic strategies for achieving clinical remission of DED encompass artificial tears and anti-inflammatory agents.^31^ Treatment with 0.1% all-trans-RA has been reported to effectively promote corneal epithelial defects,^32^ whereas 0.005% RA has demonstrated the ability to reverse corneal keratinization in xerophthalmic rabbits.^33^ Clinical studies have shown that vitamin A eye drops significantly improve DE symptoms and enhance corneal epithelial repair in patients.^34^^–^^36^ The pathogenesis of corneal epithelial-associated DE, however, remains relatively obscure. The key finding of this study was that the aberrant metabolic pathway of RA in *Aqp5^−/−^* mice resulted in a decrease in cell proliferation and an increase in apoptosis of CECs.

The aquaporin family plays a pivotal role in maintaining water permeability and facilitating transmembrane H_2_O_2_ transport.^6^^,^^7^^,^^37^ Among the various ROS, the accumulation of H_2_O_2_ serves as a central mediator in stress signal transduction pathways.^38^ The presence of Aqp5 has been reported to stimulate the antioxidant system and activate transcription factors triggered by H_2_O_2_ osmotically. This stimulation enhances the production of antioxidant defense, facilitating the restoration of cellular redox reaction equilibrium, repair of oxidized biomolecules, and promotion of ROS removal.^39^ The corneal epithelium is a tissue characterized by strong expression of Aqp5.^40^^,^^41^ Through the current investigation, it has been revealed that Aqp5 plays a crucial role not only in water transport but also in maintaining corneal stability, promoting wound healing,^13^ and regulating cellular processes, such as adhesion,^42^ migration, proliferation,^43^ and differentiation.^44^ Our previous research has demonstrated that Aqp5 regulates the homeostasis and functionality of CECs by modulating intracellular levels of ROS, thereby influencing the Wnt/β-catenin signaling pathway.^21^ It has been reported that ROS levels play a crucial role in regulating the nuclear translocation of AP-1, which in turn controls various pro-inflammatory genes.^45^ The role of JunB, a member of the AP-1 transcription factor family, as a redox-sensitive transcription factor activated in response to mitochondrial redox imbalance has been elucidated in skin fibroblasts. However, there is no evidence suggesting that Aqp5 affects JunB expression in the corneal epithelium. In this study, we have discovered that deletion of Aqp5 leads to alterations in the ultrastructure of CECs, characterized by larger intercellular gaps and reduced tight junctions, as well as increased apoptosis and punctate abnormalities. WB and transcriptome sequencing analyses demonstrated a notable increase of JunB expression in the corneas of *Aqp5^−/−^* mice.

The RA metabolic pathway is closely associated with the function of the corneal epithelium. The crucial rate-limiting enzyme Aldh1a1 oxidizes retinal to RA, which binds to extranuclear Rarα for signal transmission.^46^ In the corneal epithelium of *Aqp5^−/−^* mice, there is a significant decrease in Aldh1a1 expression, regulated by multiple factors. No reports have indicated that Aqp5 knockout leads to DE through RA abnormalities.

In this study, we have identified that JunB directly binds to the promoter region of Aldh1a1 and exerts a negative regulatory effect on its expression. Upon treatment with SR, primary *Aqp5^+/+^* mouse CECs exhibited significantly increased levels of Aldh1a1. Furthermore, administration of RA or SR to *Aqp5^−/−^* mice resulted in partial reversal of the DE phenotype by promoting cell proliferation and inhibiting cell apoptosis. When combined, these results imply that canonical Aqp5 facilitates RA synthesis through JunB-mediated mechanisms, thereby governing corneal epithelial homeostasis and regeneration.

To further elucidate the role of Aqp5 and RA in the DE process, we subjected HCECs to hypertonic NaCl treatment to establish an in vitro DE model and treated them with AZA, an aquaporin inhibitor, to simulate Aqp5’s deletion. Genes linked to the RA pathway and Aqp5 both showed decreased expression in NaCl-induced HCECs. Treatment with RA or SR significantly reduced apoptotic cell numbers and expression of apoptosis-related proteins in HCECs, whereas enhancing their proliferation ability when exposed to NaCl or AZA, thus confirming our findings from in vivo experiments.

In conclusion, our study has validated the pivotal role of AQP5 in CECs. We have provided detailed explanations on how AQP5 influences the transcription factor JunB, which subsequently regulates RA levels, cell proliferation, and apoptosis specifically in the corneal epithelium. Our findings suggest that targeting AQP5 could potentially offer therapeutic opportunities for treating corneal diseases.

However, it is important to acknowledge the limitations of this study that need to be taken into consideration. First, the direct target of the RA anti-apoptotic effect was not investigated. Second, in related in vitro experiments simulating AQP5 knockout, a more appropriate choice would be to select HCECs specifically knocking out AQP5. Despite these shortcomings, our findings highlight the significance of AQP5 in DE and demonstrate the therapeutic potential of RA intervention. Future research should focus on translating these findings into clinical interventions and exploring the broader implications of AQP5 dysregulation in ocular pathologies.

## Supplementary Material

Supplement 1

Supplement 2

Supplement 3

Supplement 4
