# Supplementary figures and images for: Reticulated Retinoic Acid Synthesis is Implicated in the Pathogenesis of Dry Eye in Aqp5 Deficiency Mice
Source: Invest Ophthalmol Vis Sci. 2024 Jul 17;65(8):25. doi: 10.1167/iovs.65.8.25 (PMC11262545; doi:10.1167/iovs.65.8.25)

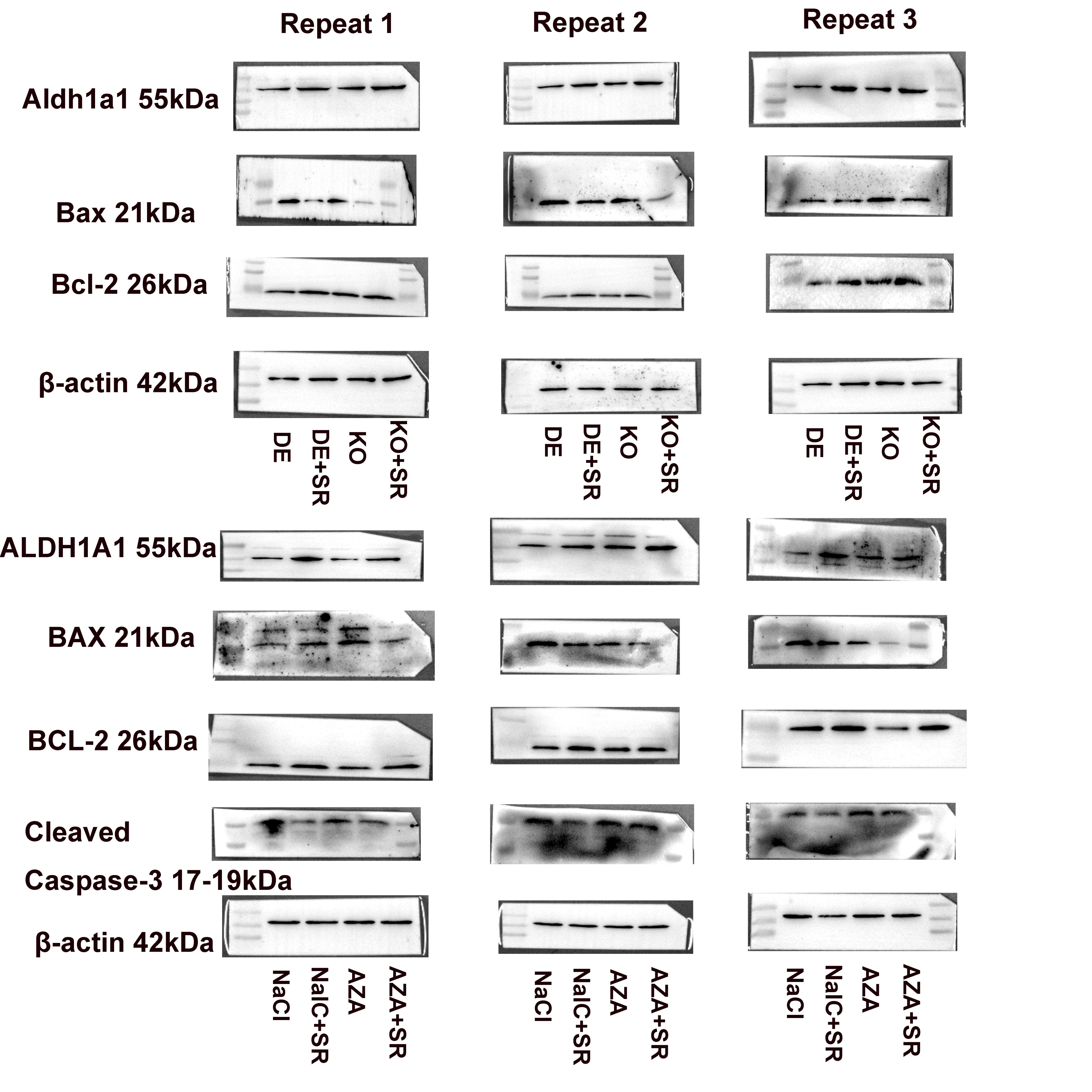

Supplement: Supplement 2 [file iovs-65-8-25_s002.pdf]

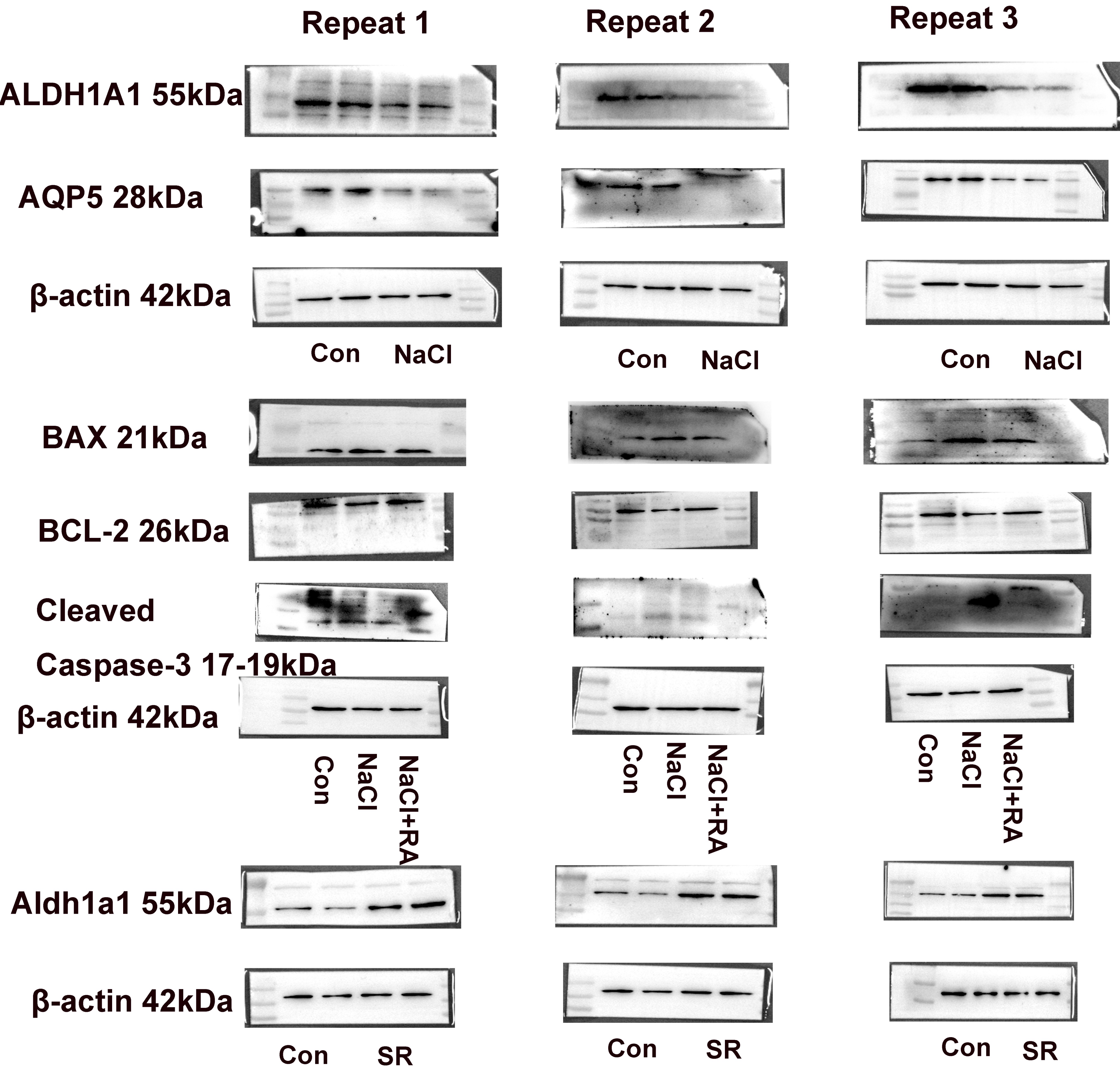

Supplement: Supplement 3 [file iovs-65-8-25_s003.pdf]
