# Supplementary material for: Reticulated Retinoic Acid Synthesis is Implicated in the Pathogenesis of Dry Eye in Aqp5 Deficiency Mice
Source: Invest Ophthalmol Vis Sci. 2024 Jul 17;65(8):25. doi: 10.1167/iovs.65.8.25 (PMC11262545; doi:10.1167/iovs.65.8.25)
Supplement: Supplement 4 [file iovs-65-8-25_s004.pdf]

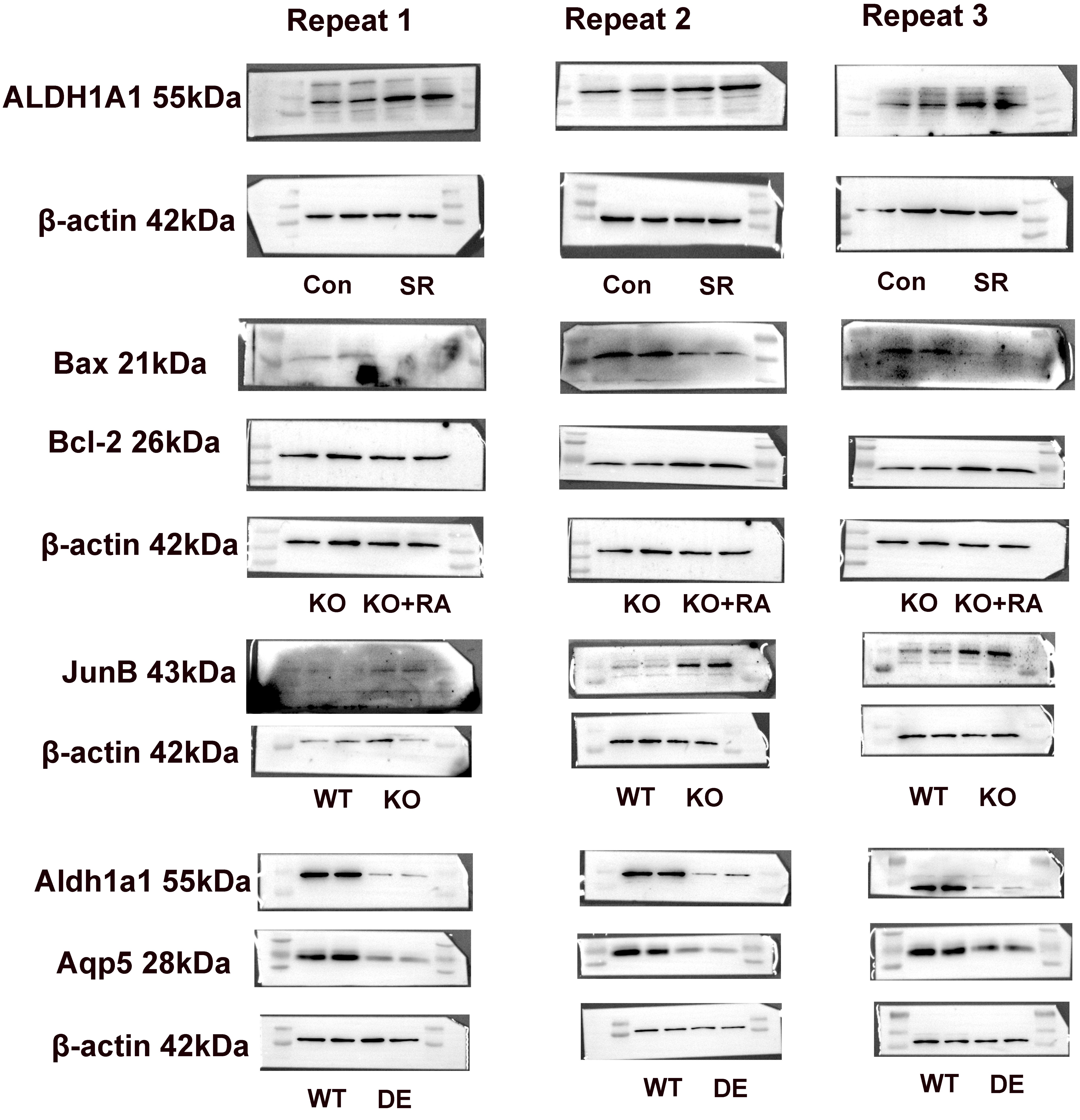
Repeat 1
Repeat 2
Repeat 3
ALDH1A1 55kDa
β-actin 42kDa
Con
SR
Bax 21kDa
Bcl-2 26kDa
β-actin 42kDa
KO
KO+RA
JunB 43kDa
β-actin 42kDa
WT
KO
Aldh1a1 55kDa
Aqp5 28kDa
β-actin 42kDa
WT
DE
